# Looking through the Glass Ceiling: A Qualitative Study of STEM Women’s Career Narratives

**DOI:** 10.3389/fpsyg.2017.00236

**Published:** 2017-02-20

**Authors:** Mary J. Amon

**Affiliations:** Developmental Cognitive Neuroscience Laboratory, Department of Psychological and Brain Sciences, Indiana University Bloomington, Bloomington, INUSA

**Keywords:** STEM women, leadership, Photovoice, career strategy, gender roles, organizational policy, Participatory Action Research, underrepresentation of women

## Abstract

Although efforts have been directed toward the advancement of women in science, technology, engineering, and mathematics (STEM) positions, little research has directly examined women’s perspectives and bottom-up strategies for advancing in male-stereotyped disciplines. The present study utilized Photovoice, a Participatory Action Research method, to identify themes that underlie women’s experiences in traditionally male-dominated fields. Photovoice enables participants to convey unique aspects of their experiences via photographs and their in-depth knowledge of a community through personal narrative. Forty-six STEM women graduate students and postdoctoral fellows completed a Photovoice activity in small groups. They presented photographs that described their experiences pursuing leadership positions in STEM fields. Three types of narratives were discovered and classified: career strategies, barriers to achievement, and buffering strategies or methods for managing barriers. Participants described three common types of career strategies and motivational factors, including professional development, collaboration, and social impact. Moreover, the lack of rewards for these workplace activities was seen as limiting professional effectiveness. In terms of barriers to achievement, women indicated they were not recognized as authority figures and often worked to build legitimacy by fostering positive relationships. Women were vigilant to other people’s perspectives, which was costly in terms of time and energy. To manage role expectations, including those related to gender, participants engaged in numerous role transitions throughout their day to accommodate workplace demands. To buffer barriers to achievement, participants found resiliency in feelings of accomplishment and recognition. Social support, particularly from mentors, helped participants cope with negative experiences and to envision their future within the field. Work-life balance also helped participants find meaning in their work and have a sense of control over their lives. Overall, common workplace challenges included a lack of social capital and limited degrees of freedom. Implications for organizational policy and future research are discussed.

## Introduction

Research on the underrepresentation of women in science, technology, engineering, and mathematics (STEM) often focuses on top-down factors that influence recruitment, retention, and promotion. Such top-down factors tend to overlook women’s unique perspectives and strategies. Women are agents of their own career success, with their own complex perceptions and bottom-up strategies within the workplace. Granting that individual experiences in the workplace give rise to personal narratives, common themes are likely to emerge across STEM women’s experiences. The goal of this research is to examine the career narratives of STEM women, or the spoken account of their experiences pursuing leadership positions in STEM. Photovoice, a Participatory Action Research method, informed by a grounded theory perspective, was used to identify the barriers that STEM women perceive as especially challenging, as well as their bottom-up approaches for managing barriers to achievement.

Women prepare for college degrees in STEM at approximately equal rates as men. However, after matriculating into college, women are less likely to pursue degrees in these fields (Hill et al., 2010). While women are more likely than men to earn a bachelor’s, Master’s, or doctoral degree, they remain the minority of degree-earning STEM students (United States Census Bureau, 2010). This is particularly true for more advanced degrees, where graduation rates in STEM favor men 2.5:1 (National Science Foundation, 2015a). Women are excluded from STEM despite generally high levels of academic achievement. In high school, girls and boys take approximately equal credits in STEM fields, with girls earning higher grades on average (Shettle et al., 2007). In higher education, women earn better grades than men and are more likely to achieve post-secondary degrees at all levels (Buchmann and DiPrete, 2006; United States Census Bureau, 2010). Despite generally high levels of achievement, women who are proficient in math-intensive fields are more likely to choose careers outside of STEM and leave STEM careers as they advance in their education (Ceci et al., 2009).

Gender discrepancies become more pronounced at the professional level, a pattern that is evidenced across both academia and industry (Trower and Chait, 2002). Women account for nearly half of the United States workforce, but compose less than 30% of the positions in STEM (National Science Foundation, 2015b). STEM women advance more slowly and are more likely to leave their positions than male peers (Valian, 1999). Overall, the higher the rank in STEM the less likely it is to be occupied by a woman, making women particularly underrepresented in leadership positions.

The shortage of women from high-ranking positions is not exclusive to STEM fields. For example, while the average corporate board has 8.8 members, 36% of companies do not have any women on their board of directors and only 8% of boards have three or more women (Gladman and Lamb, 2012). However, the shortage of women is particularly striking when leadership and STEM intersect: at 61%, energy companies have the highest percentage of boards with no women, and in academia, only 31% of full-time STEM faculty and 27% of deans and department heads are women (National Science Foundation, 2015b).

Despite evidence that attrition of women from STEM disciplines increases as women progress through college, graduate school, professional, and leadership ranks, surprisingly little research has been conducted on the intersection between STEM and leadership (McCullough, 2011). In order to address this problem, it is essential to understand features of the workplace climate that are objectionable and unwelcoming to women. Qualitative research provides a unique opportunity to synthesize the complex experiences of STEM women by identifying common themes that underlie women’s career narratives. To date, a small number of qualitative studies with student participants have highlighted the importance that STEM women place on social support, coursework success, and early and positive exposure to STEM disciplines (e.g., Hughes, 2010; Packard et al., 2011). For example, STEM women transitioning from a community college to a 4-year program identified social support in the form of helpful academic advisors and professors as significant resources for overcoming obstacles such as poor course experiences and limited finances (Packard et al., 2011). A study by Riffle et al. (2013) highlighted the spoken accounts of STEM faculty using semi-structured interviews. Men and women faculty members identified aspects of their work environment that facilitated success, including mentorship, social support, and work-life balance. However, there were gender differences in perceptions of departmental climate. Unlike men, women reported greater discrimination and sexism during interviews, less departmental collegiality, and holding less influence in their department. In addition, though men and women participants were found to have equal levels of productivity, women noted that their departments viewed their productivity as lower than their male counterparts (Riffle et al., 2013). Qualitative studies such as these lay the foundation for accounts of workplace gender inequality by beginning to draw attention to factors that are perceived as major obstacles and supports in pursuing STEM careers.

The present study expanded on this work by using Photovoice to examine the career narratives of STEM women graduate students and postdoctoral fellows pursuing leadership positions. Photovoice (Wang and Burris, 1997) is a method of group analysis that requires participants to take photographs that represent their viewpoint and present them during a group discussion. Participatory Action Research methods such as Photovoice recognize *experiential learning* as a legitimate source of knowledge and allow researchers to gather information about the targets’ perspective. Photovoice is particularly well-suited for research on STEM women, as it was developed based on feminist theory and literature on critical consciousness (Wang, 1999). As defined by Weiler (1988), feminist methodology is characterized by an emphasis on women’s subjective, everyday experiences. Critical consciousness literature argues that people’s perspectives can be used to break a culture of silence and increase awareness of social issues (Freire, 1973). Photovoice was used to promote a critical dialog about how women’s everyday experiences in male-dominated careers impact broader gender distribution. This approach is useful in synthesizing the complex issues that influence STEM women’s career trajectories: First, as a consequence of identifying experiences common to STEM women, this approach is positioned to consolidate a number of topics relevant to the advancement of women in STEM (e.g., organizational climate and incentive structure). Second, Photovoice has direct ties to policy advocacy by providing a platform for STEM women to identify aspects of the work environment that they perceive as especially relevant to their career trajectories, including changes that need to be made to enhance their success. Third, qualitative research can facilitate the identification of new research directions useful for understanding and enhancing STEM women’s career advancement.

The present study expands on previous qualitative studies of women’s experiences in STEM in three key ways. First, STEM women graduate students and postdoctoral fellows were recruited from STEM leadership workshops, representing a group of women experienced in STEM and with explicit interest in leadership positions. Women in this career stage are making critical decisions about their career trajectory, with many opting for careers in education or healthcare over STEM occupations (Beede et al., 2011). Thus, participants have a unique vantage point as they are actively gaining insight into the advantages and disadvantages of STEM careers and navigating their career path. Second, this is the first study using Photovoice to examine STEM women’s experiences and the first qualitative study to focus on STEM women in leadership. Photovoice gives participants the opportunity to identify and share aspects of their experiences that are most important to them and is more open-ended than methods like structured or semi-structured interviews. Literature on STEM women emphasizes a wide range of factors that influence women’s career outcomes, from implicit stereotype cues to organizational policies (e.g., Valian, 1999; Smith and White, 2002; Murphy et al., 2007; Bilimoria and Lord, 2014). However, it remains unclear which of these aspects of the work environment that women find more or less challenging. Identification of themes underlying women’s experiences in STEM via qualitative methods can be used to highlight their observations and perceptions of the field in a way that is atypical of most experimental research. Third, the present research recruited a relatively large and diverse group of participants. Forty-six participants completed the Photovoice exercise, which is considerably more than the median of 13 participants in Photovoice studies (Catalani and Minkler, 2010). The sample also included 16 international participants, representing 11 different countries.

Science, technology, engineering, and mathematics women graduate students participating in leadership workshops were invited to a an additional workshop session where they had the opportunity to present four photographs depicting their experiences with STEM leadership and discuss them in a small group setting. Specifically, participants were asked to share with a group two photographs depicting the past experience in STEM and two describing their future in STEM. Transcripts from the eight workshops sessions were coded borrowing from grounded theory framework (Glaser and Strauss, 1967). The grounded theory perspective forwarded by Strauss allows for basic research questions and predictions to guide analysis (Corbin and Strauss, 1990; Devadas et al., 2011). Accordingly, it was hypothesized that STEM women would acknowledge the effects of gender stereotypes in STEM, reflecting their common experience with gender stereotypes. Additional coding was carried out inductively to identify emergent themes in the raw data. The current article discusses the career strategies, barriers to achievement, and approaches for managing barriers to achievement described by STEM women, as well as implications for research and practice.

## Materials and Methods

### Participants

Forty-six participants from a leadership workshop for STEM women enrolled in a second session in order to complete the Photovoice activity. Participants signed up for one of eight possible workshop sessions, with an average of six participants in each session. Participants from the final sample ranged in age from 21 to 51 years (*M* = 29, *SD* = 6.13). Thirty-five percent of the sample was international students, with participants representing 11 different countries. Sixty-four percent of participants identified as White, 22% Asian, 4% Black, 4% Hispanic, 4% Biracial, and 2% Native American. A variety of fields were represented from the natural sciences (50%), medicine and health (28%), and engineering (22%).

### Procedure

Women graduate students and postdoctoral fellows in STEM fields from a large public research university were recruited to participate in leadership workshops via flyers and e-mails. Participants indicated their availability for eight different workshop groups. Whenever possible, they were matched into groups in order to have individuals from different academic disciplines in each session. Upon arriving to the first workshop session, participants were asked to sign an informed consent and complete a demographic survey. The first session included interactive activities designed to identify leadership role models and personal values, develop personal action plans, and practice conflict resolution. Prior to the end of the workshop, participants were invited back for a second session to complete the Photovoice activity.

In line with the goal to understand the broad range of issues related to women’s advancement in STEM, Photovoice allowed for open-ended discussion about participants’ experiences in STEM. Participants were asked to prepare two photographs describing past experiences with leadership and two pictures representing their future leadership aspirations. Participants were invited to take their own pictures or use images found online. Directions did not prompt participants to attend to a particular issue. Photographs were e-mailed to the researcher prior to workshop sessions in order to format them into a slide show for the Photovoice discussion. Audio and video equipment was positioned to record each workshop session. The Photovoice activity was transcribed, producing 80 single-spaced pages of transcription.

The Photovoice activity was held 1 week after the participants’ original workshop sessions. Participants were invited to discuss their Photovoice pictures with the group, and participants determined the order of presentation (i.e., who presented and the order of photographs). Each individual projected their photographs onto a large screen at the front of the room and explained the meaning of each of their four photographs. After each individual presented a photograph, the workshop facilitator (i.e., a female graduate student) or participants were allowed to ask questions or comment. For example, a participant projected a picture of their messy desk while explaining how it relates to their past experience pursuing STEM leadership, in this case, noting that they juggle many projects and do not always have a healthy lifestyle due to high workplace demands. Other participants related to this narrative, noting that they too do not always have a healthy lifestyle or work-life balance even though they consider those things important. Within this structure, the workshop facilitator and participants were able to probe further comments. Individual narratives can elicit agreement or disagreement from peers as they discuss photographs in a small group setting, providing information about the typicality of a given experience. All research was carried out in accordance with the protocol approved by the University of Cincinnati’s Institutional Review Board.

### Data Analysis

The grounded theory approach to qualitative analysis forwarded by Glaser and Strauss (1967) provides a framework for text analysis. Grounded theory utilizes a constant comparative method whereby text is assigned to a category based on content and compared with other text included in the same category (Glaser and Strauss, 1967). Categories are created if text needs to be further differentiated, or if categories need to be integrated. Over the course of the analysis, categories are arranged into a hierarchy that is representative of their relationship to one another. This method of coding is used to uncover the full range of categories possible, their dimensions, the conditions under which it is pronounced, its consequences, and its relationship to other categories. Unlike many experimental studies that typically examine cause-and-effect relationships, qualitative research is often utilized at the discovery stage of research in order to develop new and testable theories.

Qualitative data was analyzed using QSR International’s NVivo 10 software to aid in examining text and coding transcripts. The author and a trained research assistant independently coded the transcripts, condensing language into categories. Categories were defined using open coding, examining the transcripts line-by-line, allowing for patterns and categories to emerge through observation (Glaser and Strauss, 1967). Text was coded when it was identified as meeting specific criteria relevant to a categorical definition. To investigate the *a priori* research questions, additional coding examined career challenges and strategies. Category labels and definitions were shared between researchers, though they were blind to the text included within each category by the other researcher. When possible, categories were collapsed into higher-order categories based on researcher consensus until no new categories or sub-categories were identified. This is an indicator of theoretical saturation, such that additional data collection and coding is unlikely to identify new emerging themes (Locke, 2001; Kreiner et al., 2009).

After categories were independently coded, they were discussed in meetings to finalize coding (Kreiner et al., 2009). Consensual validation was used to refine category definitions and reduce bias (Kreiner et al., 2009). Agreement was reached on categories and content by the researchers and coding was adjusted. Only categories mentioned by 25% or more participants were included as themes (Dutton and Dukerich, 1991). Relationships across categories were then examined using axial coding, rendering nine primary themes (Strauss and Corbin, 1998). After identifying the most prominent thematic connections between categories and subcategories, three broader frameworks were identified using selective coding (Strauss and Corbin, 1998). These frameworks and their underlying themes were examined and adjusted by six study participants in order to verify its trustworthiness (Creswell, 2007). Three additional faculty-level researchers were also enlisted to review the findings for alternate themes and explanations (Lincoln and Guba, 1985; Creswell, 2007). Participants’ descriptions of past experiences and future aspirations in STEM leadership were coded for themes across groups. Three complementary frameworks were identified from the participants’ Photovoice narratives: Career motivation, barriers to participation, and buffering strategies. The conceptual frameworks and underlying themes are elaborated below (see **Table 1** and **2**).

**Table 1:** Summary of themes and theoretical significance.

Theoretical framework	Theme number	Theme	First-order codes
Motivation	1	Collaboration	Work with one or more people toward a common goal.
	2	Social impact	Activities or goals that have social value and affect the surrounding community.
	3	Self-development	Work to advance personal skills and potential.

Barriers	4	Lack of authority	Lack of power or right to make decisions, influence, or enforce obedience.
	5	Vigilance	Monitoring environmental and interpersonal cues.
	6	Gender stereotypes	Generalizations about gender differences and roles.

Buffers	7	Accomplishment	Positive experiences or recognition.
	8	Social support	Positive social engagement that enhances psychological resources or leads an individual to believe that they are valued and accepted.
	9	Work-life balance	A comfortable balance between professional work and personal lifestyle.

**Table 2:** Illustrative evidence for themes.

Theme	Illustrative quotations
Collaboration	“It was sort of like a musical kind of harmony, where somebody is playing ‘the lead’ obviously but then the backup is just as important.”
Social impact	“I hope to be a leader amongst my peers professionally as well as in the community.”
Self-development	“I want to improve myself, to get more.”
Lack of authority	“I can give you a token. I can call you up and recognize you for your work and thank you. That’s all I have. That’s the only power.”
Vigilance	“We haven’t really butted heads with anybody or really said a whole lot…you just kind of listen and deal with it.”
Gender stereotypes	“She’s either going to be an authoritative b-word, or she’s going to be like this motherly figure.”
Accomplishment	“But the whole cause itself is really, really great. And I think it was a really good way for me to connect teaching, mentoring, and science.”
Social support	“So I got a really good friend, good mentor, a very supportive family.”
Work-life balance	“Even if it’s 10 min [off of work] it shows that you’re in control and you know what you want to get out of this.”

## Results

### Framework 1: Motivation

In describing past experiences and future pursuits of “leadership,” participants had a broad definition of leadership that extended beyond managing others to the achievement of individual and group goals. In terms of individual goals, STEM advancement was seen not only as a way to achieve career success, but also personal development (*Theme 1*): “Leadership is a good way to help me to improve myself…I don’t want to just stay in one specific level.” Leadership was also seen as a way to help others achieve their goals and develop positive relationships. As one participant put it, “I try to find a sky for me to fly, and…if I want to be a leader I also have to find a sky for the others.”

Women noted a number of methods important to facilitating effective collaboration (*Theme 2*). Participants believed that collaboration could be enhanced through clear communication, justification of team goals, and individual recognition. Participants noted the importance of actively motivating colleagues, as exemplified by one participant’s statement: “Appreciating their work and being respectful of them is really important; not treating them like your workers and giving them menial tasks but making them feel valuable to the project.” The value of working side-by-side with others was seen as a strategy for teaching other people positive work habits, and demonstrated participant’s preference for a more egalitarian work environment. Overall, participants described their leadership style as transformational (Burns, 1978), where leaders work to motivate team members toward the achievement of a common goal.

Participants sought to develop a full range of skills within the workplace to influence others in a meaningful way. Teaching, mentorship, service, organizational, and applied work were all seen as opportunities for broader impact (*Theme 3*). Among those pursuing careers in research, their goals were often geared toward promoting the well-being of others (e.g., water conservation, disease control, and social programming). Other participants questioned how much impact research has in comparison to applied work. One woman stated that while she had never discussed career options within her department, she was strongly considering going into applied work, “I don’t want to belittle it by saying ‘cookie-cutter’ or saying this is the ‘typical route’ that graduate students take, to be a professor. And that ignores the need for better science education at the lower levels.” Whether interested in research or applied work, participants engaged in big picture thinking: Women not only desired to advance in their field, they saw leadership as a means of self-actualization (*Theme 1*), collaboration (*Theme 2*), and social impact (*Theme 3*).

### Framework 2: Barriers

The second framework outlined the circumstances under which goal achievement became especially challenging. Participants generally preferred a transformational leadership style, but they did not always feel empowered as leaders. One participant noted, “…this is literally kind of like a struggle, not just finding leadership opportunities, but once you’re in them I feel like I am kind of up against somebody most of the time.” When women were in authority positions, they did not assume that subordinates would take their direction seriously and instead worked to build legitimacy by fostering positive relationships. The “fair-and-balanced” approach some women desired may have, in part, reflected their uneasiness in positions of authority (*Theme 4*). As one woman stated, “I can give you a token. I can call you up and recognize you for your work and thank you. That’s all I have. That’s the only power.” Participants were primarily focused on increasing their social status through relationships, rather than increasing their personal power or access to resources (e.g., Sachdev and Bourhis, 1991).

Attempting positive relationships and social impact were costly in terms of time and energy. To support a positive and collaborative work environment, women were vigilant to other people’s perspectives (*Theme 5*). Participants frequently noted the thoughts and feelings of others during their narratives. One participant stated, “We should always do well in our own business, but we also need to think about others, be considerate, and so everyone can be comfortable.” Participants were also vigilant to how their own behavior might be evaluated by others. One participant described carefully watching her steps, “I’m walking around with my shoes untied. I always have to look and make sure I’m not going to trip and fall.” She contrasted this with her “comfy shoes” that she wore at home. Another participant stated the importance of controlling other people’s impressions of her, “Wear your dark-colored suit, flat shoes, and no jewelry.”

Monitoring social interactions was particularly challenging when gender dynamics were involved (*Theme 6*). Participants noted the dichotomy between being the “motherly figure” and the “authoritative b-word,” and sometimes felt the need to adapt their leadership style to the situation. A woman with industry experience stated, “I always work in a man environment, so I cannot be too soft. They just crush you.” She contrasted this with working with women, explaining, “It’s like if you’re in this as equals, then they think you’re not in it to get anything. Somehow you have to keep your feminine, soft side.” When working with men, participants felt the need to mask emotions and appear confident, but among women they tried to act less threatening, more egalitarian, and attentive to emotion. Some women felt that situational pressures could be overcome by adopting a more individualized leadership style, and other women expressed their continuing journey to find a leadership identity. Upon hearing other participants discuss how they changed their behavior based on context, one woman noted, “All I can do is be a good person and be strong in what I do.”

### Framework 3: Buffers

The third framework illustrated coping strategies participants used to buffer against career challenges they encountered. Participants sought comfort in their achievements (*Theme 7*). Feelings of accomplishment reassured women that they were on the right career path. Participants often noted achievements in mentoring, service, and applied roles. In these positions women were more readily elevated into leadership, developed relationships with others, and gained respect. One woman noted about her teaching, “I’ve had great success with being a leader in that people really appreciate me and I have gotten really good feedback.” For some women, finding comfort in their achievements meant being appreciative of their current positions. As one participant stated, “Whatever you do and what role you are in in your future career, even though it can be boring or a simple role, as long as you have positive thinking and you are smiling you can do this job very well.” This perspective was controversial among other participants, some of whom argued that women are too easily satisfied with *just* having a job, rather than expecting to be treated fairly based on their qualifications.

Outside of their own accomplishments, participants found resiliency in social support offered by mentors, friends, and family (*Theme 8*). The encouragement of mentors helped them cope with negative experiences, and to envision their future within the field—as one participant said, “I do so many things now and I attribute it to my advisor giving me so many opportunities and just kind of encouraging me.” However, the difficult balance between the ideal woman and ideal leader led to a lack of real world role models for some participants. For example, one participant stated, “I have role models for leadership, and I have role models for personal growth, but both of them together they don’t really exist. And it’s hard to even imagine a real, tangible opportunity.” Perhaps because of this, some women adopted an informal definition of ‘mentorship,’ looking for guidance from coworkers, friends, and professionals outside of their field. Mentorship outside of formal supervision helped women navigate the politics of their fields.

Family was another important source of support and an arena where many women took on a leadership role. Participants discussed future leadership aspirations in terms of family, as numerous women considered motherhood as a significant leadership role. As one women explained, “I would like to become a mother someday. So that’s another big kind of leadership thing that will be hard to balance.” Work-life balance also helped women find meaning in their work and have a sense of control over their lives (*Theme 9*). One woman described the importance of taking time off work saying, “Even if it’s 10 min, it shows that you’re in control and you know what you want to get out of this.”

## Discussion

The present study is the first to qualitatively examine the experiences and perspectives of STEM women using Photovoice as a Participatory Action Research method. Findings provide a basis for better understanding the complex ways that gender stereotypes surface in organizations, as well as the bottom-up strategies STEM women employ to cope with workplace challenges. Specifically, results provide a framework for understanding women’s work preferences, challenges, and buffering strategies. In line with its history as a advocacy tool, Photovoice also generated insight into policies that can best support and enhance STEM women’s career success.

While institutional policies were mentioned more or less indirectly, the frequency and depth of narrative surrounding interpersonal interactions suggests that this is often the most immediate and troubling aspect of the work environment for women. Women were mindful of other people’s perspectives and actively worked to manage them. Participants described being held accountable for multiple, often conflicting, roles in the workplace related to gender and STEM. Women’s perceptions of conflicting expectations, between acting “soft” and “hard” as they put it, is consistent with literature on role congruity and the evaluation of women in typically male-dominated fields. Role congruity theory states that, because men have traditionally occupied positions in science and leadership, career success in these fields are associated with masculine traits (Eagly and Karau, 2002). As a result, women are viewed as unfit for STEM, particularly in STEM leadership, and are also evaluated more negatively when occupying these roles. Women are viewed as less likely to succeed, less likely to be promoted, and less likely to become a leader when in male-dominated sectors than when in female-dominated professions (Garcia-Retamero and Lopez-Zafra, 2006). Women who succeed in spite of these stereotypes often experience backlash for stepping outside of their prescribed social role. For instance, women in senior management typically have less authority, less opportunity for advancement, and receive fewer rewards than their male peers (Jacobs, 1992). At first glance it might appear that STEM women are too image-focused and work unnecessarily hard to manage their interpersonal interactions, but these efforts may be a reaction to the negative evaluations commonly encountered by STEM women.

Women in this study responded to perceived gender role conflict with two primary strategies: First, some women adopted distinct behaviors within different contexts, remaining vigilant to cues regarding role expectations. This workplace strategy required role transitions throughout their day to accommodate workplace demands (cf. Schein, 1971; Van Maanen, 1982). Role transitions entail “the psychological (and, where relevant, physical) movement between roles, including disengagement from one role (role exit) and engagement in another (role entry; Burr, 1972; Richter, 1984)” (Ashforth et al., 2000). Each transition requires psychological preparation, as roles require varying levels of attention and arousal (Ashforth, 2001), and may therefore be costly in terms of cognitive and physical resources. Individuals vary in the extent to which their roles are segregated from one another (Ashforth, 2001); the more integrated roles are, the less challenging it is to transition between them. Along these lines, some women adopted a second workplace strategy and avoided numerous role transitions by adopting an individualized leadership style that they could comfortably apply across a variety of situations. Given the relatively large age range of participants, it is possible that older participants were more likely to have developed a stable sense of self and were less easily influenced by social pressures.

In addition to the interpersonal demands reported by STEM women, women often described being socially disconnected from both leadership and subordinates. Consistent with research that women are viewed as less competent leaders (e.g., Jacobs, 1992; Garcia-Retamero and Lopez-Zafra, 2006), even when women were in leadership positions, they noted they were not treated as authority figures. Participants appealed to subordinates by fostering positive relationships, working alongside them, or incentivizing them with rewards. Women also had a difficult time identifying mentors and role models who represented, not only a desirable career path, but also a desirable lifestyle. A lack of real world examples meant that women had a difficult time imagining how they would be able to succeed in STEM.

Women’s focus on social interactions, vs. policy, as barriers to achievement suggests that women are more frequently and directly confronted with the former, and that these challenges are viewed as more personal in nature. The focus of narratives on social interactions may reflect a shifting tide where organizational policy may change over time to support more gender-neutral practices. Even so, gender stereotypes, societal norms, and organizational climate are more enduring and difficult to change. As one women stated, “Just because the policy changes does not mean [people’s] beliefs change.” Overall, women reported a lack of *social capital*, or “the aggregate of the actual or potential resources which are linked to possession of a durable network of more or less institutionalized relationships of mutual acquaintance or recognition” (Bourdieu, 1985, p. 248). Women devoted significant time and energy to fostering group cohesion and developing professional relationships: Monitoring social cues, accommodating social expectations, implementing strategies to enhance collaboration, and pursuing applied work and social impact all require significant effort. These efforts were not always rewarded, as women struggled to establish relationships with subordinates and mentors. Women also noted that they faced negative evaluation, from being belittled during presentations to being harshly questioned for their career decisions. Similar reports come from the faculty-level, as women professors generally report low-levels of collegiality (Riffle et al., 2013). Despite committing significant resources to maintaining positive relationships, women often failed to receive benefits from participation in groups (Portes, 1998).

Science, technology, engineering, and mathematics women encountered two distinct limitations in the workplace that restricted their social interactions and professional activities. First, women’s workplace behavior was restricted by the potential for negative evaluation and its implied consequences. Women worked to overcome negative evaluation by maintaining a certain appearance, adopting different mannerisms based on role expectations, working to make sure people “liked” them, and monitoring their behavior for what could be perceived as “mistakes.” Women felt that they were encouraged, implicitly or explicitly, to mold their behavior to avoid negative social evaluation. Second, women were limited by an incentive structure that rewarded a relatively narrow range of professional activities. Numerous women reported that their departments valued basic research over applied directions, and the pressure on academics to publish and pursue research trajectories without direct application caused some participants to question if their goals aligned with a career in STEM, vs. fields like health or education. Opportunities for self-development, collaboration, and social impact were identified as major motivational factors for STEM women—a lack of rewards for these activities may therefore hinder STEM women’s professional advancement. The space that women in STEM are allowed to occupy is enclosed by narrow boundaries and is enforced by the potential for negative social evaluation and career stagnation. Compared to their male peers, STEM women have *fewer degrees of freedom* in the workplace.

### Policy Implications

Though women’s narratives often referenced the importance of interpersonal interactions to shaping their careers, institutional policies can foster a workplace climate conducive to collegiality and increased opportunity for a diverse faculty. The threat of negative evaluation significantly impacts women’s daily activities. STEM leadership may benefit from an awareness of the chronic judgment that women are often subject to by both female and male coworkers and subordinates. Methods of formal evaluation used by departments and universities can be altered or weighted to take into account gender biases typical of student and departmental evaluations (Kaschak, 1978; Sprague and Massoni, 2005; Moss-Racusin et al., 2012). Departments can also reduce the threat of negative evaluation and increase women’s social capital by promoting diversity and a positive workplace climate. In particular, institutions can explicitly advocate for workplace collegiality, offer structured networking opportunities, institute faculty mentorship programs along with mentorship training, and incentivize departmental and interdepartmental collaboration.

The ability to recruit, retain, and promote a diverse workforce hinges on the ability of an organization to value heterogeneous perspectives and contributions. In this study, numerous participants noted the value that they placed on applied scholarship. At an institutional level, criteria for promotion and tenure can be restructured to reward a more diverse set of workplace activities. For example, some women may place greater emphasis on applied work over publishing, the former of which can be explicitly incentivized. In addition, recognition for achievements, in the form of competitive grants and awards, can advance women’s research and publicly recognize their achievements. Individuals who experience barriers to participation in STEM need sufficient reason to enter into and persevere within these stereotyped domains. Women can be encouraged to remain in STEM with opportunities for self-development, collaboration, and social impact. Organizations can incentivize these activities and emphasize these opportunities through organizational messaging.

In the present study, a fulfilling personal and family life is closely tied to STEM women’s feelings of success and life satisfaction. This is consistent with research suggesting that policies promoting work-life balance are essential to recruiting, retaining, and advancing faculty in academia (Welch et al., 2011). A variety of initiatives can bolster STEM employee’s support network and work-life balance (e.g., Kelly, 1999; Association for Women in Science, 2001; Tower and Dilks, 2015): Employee benefits can be structured to allow for flexible work hours, leadership can take into account family obligations during scheduling, organizations can offer paid maternity and paternity leave, and dual-career hires can be made a greater priority, as these issues may disproportionately influence female employees.

### Future Directions and Limitations

One strength of qualitative research is its ability to formulate new research questions (Glaser and Strauss, 1967). While there is much work to be done to understand the complex array of factors that influence women’s participation in STEM, the present study generated two distinct research questions to be explored further. First, a significant portion of women in this study described their strategy for dealing with conflicting demands in the workplace, particularly disparate role expectations for women vs. scientists in male-dominated domains. Some of these women adapted their behavior to different contexts based on situational cues; other women adopted a personal leadership style that they carried across contexts. Women in the latter tended to be older and more experienced scientists. It is unclear from this study if there is an association between seniority and leadership style. Future research should investigate the perspectives of women in more or less advanced positions in STEM to examine how factors influence women’s participation in different ranks, as well as whether women in various career stages employ different strategies for dealing with workplace challenges. Along these lines, it is important to increase our understanding of which career strategies are more or less useful for overcoming barriers to achievement. Second, many participants preferred applied work over basic research. Additional research is needed to examine if this preference holds true across a broader sample. It is also essential to understand whether or not women are implicitly or explicitly encouraged by others to go into applied work over basic research. Women may be directed away from basic research, which is likely to be more male-stereotyped.

A limitation of this study is that participants did not play a significant role as decision-makers in the research project. Photovoice often emphasizes the involvement of participants in study design and implementation. However, participants retained significant independence in their personal contribution, and a number of participants were also recruited to help in data interpretation after data analysis was completed. In addition, material from the workshop women were recruited from may have cued women to talk about particular aspects of their career progression. An attempt was made to control for this possibility by making the original workshops activity-based. Overall, the workshop format was an effective recruitment method, and career narratives were largely unrelated to workshop material, which focused on values, conflict management, and role model identification.

Finally, the current study is limited in focusing on the experiences of STEM graduate students and postdoctoral fellows. For the purposes of this study, graduate women offer a unique perspective. Having persevered as an undergraduate in typically male-dominated fields and continuing into advanced training, they have made a considerable personal investment in their fields. They are also gaining a new perspective into the professional world ahead of them. As graduate women advance in STEM they remain vulnerable to the gender stereotypes that pervade these fields.

## Conclusion

Women identify interpersonal interactions as limiting their professional opportunities more often than institutional policy. In particular, women report having less social capital and fewer degrees of freedom than their male counterparts. Their reports are largely consistent with research demonstrating women’s lack of authority and the negative evaluation of women compared to men (Jacobs, 1992; Garcia-Retamero and Lopez-Zafra, 2006). The close relationship between women’s career narratives and previous research findings supports that notion that qualitative research is not only useful in understanding the perceptions of a given population, but that group analysis is relatively reliable in describing their experiences. The findings also synthesize a number of the complex issues that influence STEM women’s career trajectories.

The present work identifies strategies implemented by women to cope with organizational and interpersonal barriers to achievement. Recognition of achievements, social support, and work-life balance assured women that their efforts pursuing STEM leadership would pay off. In addition, some women managed conflicting role expectations by adapting their behavior based on context; other women adopted a more individualized leadership style that they could comfortably maintain across contexts and regardless of social demands.

Findings enhance understanding of how gender stereotypes manifest and impact women in male-dominated careers, and have a number of implications for organizational policy. By emphasizing the importance of positive interpersonal interactions and organizational climate to career success, women’s narratives indicate the importance of organizational policies that incentivize collegiality and collaboration. Though barriers to achievement often occur at an interpersonal level, a variety of organizational policies can address these challenges by promoting fair workplace evaluation, positive climate, collaboration, work-life balance, and an incentive structure that rewards a variety of scholarly activities.

## Author Contributions

MA was responsible for the research question, study design, running participants, data analysis, and manuscript.

## Conflict of Interest Statement

The author declares that the research was conducted in the absence of any commercial or financial relationships that could be construed as a potential conflict of interest.

## References

[B1] Ashforth B. E. (2001). *Role Transitions in Organizational Life: An Identity-Based Perspective*. Mahwah, N.J: Lawrence Erlbaume Associates.

[B2] Ashforth B. E. Kreiner G. E. Fugate M. (2000). All in a day’s work: boundaries and micro role transitions. *Acad. Manag. Rev.* 25 472–491. 10.5465/AMR.2000.3363315

[B3] Association for Women in Science (2001). *Association for Women in Science.* Available at: http://www.awis.org/?WorkLife

[B4] Beede D. Julian T. Langdon D. McKittrick G. Khan B. Doms M. (2011). *Women in STEM: A Gender Gap to Innovation.* Available at: http://www.esa.doc.gov/sites/default/files/womeninstemagaptoinnovation8311.pdf 10.2139/ssrn.1964782

[B5] Bilimoria D. Lord L. (2014). *Women in STEM Careers: International Perspectives on Increasing Workforce Participation, Advancement and Leadership*. Northampton, MA: Edward Elgar Publishing 10.4337/9781781954072

[B6] Bourdieu P. (1985). “The forms of capital,” in *Handbook of Theory and Research for the Sociology of Education* ed. Richardson J. G. (New York, NY: Greenwood) 241–258.

[B7] Buchmann C. DiPrete T. A. (2006). The growing female advantage in college completion: the role of family background and academic achievement. *Am. Sociol. Rev.* 71 515–541. 10.1177/000312240607100401

[B8] Burns J. M. (1978). *Leadership.* New York. NY: Harper & Row.

[B9] Burr W. R. (1972). Role transitions: a reformulation of theory. *J. Marriage Fam.* 34 407–416. 10.2307/350436

[B10] Catalani C. Minkler M. (2010). Photovoice: a review of the literature in health and public health. *Health Educ. Behav.* 37 424–451. 10.1177/1090198109342084 19797541

[B11] Ceci S. J. Williams W. M. Barnett S. M. (2009). Women’s underrepresentation in science: sociocultural and biological considerations. *Psychol. Bull.* 135 218–261. 10.1037/a0014412 19254079

[B12] Corbin J. M. Strauss A. (1990). Grounded theory research: procedures, canons, and evaluative criteria. *Qual. Sociol.* 13 3–21. 10.1007/BF00988593

[B13] Creswell J. W. (2007). *Qualitative Inquiry & Research Design: Choosing Among Five Approaches* 2nd Edn. Thousand Oaks, CA: Sage.

[B14] Devadas U. M. Silong A. D. Ismail I. A. (2011). The relevance of Glaserian and Straussian grounded theory: approaches in researching human resource development. *Int. J. Model. Optim.* 11 348–352.

[B15] Dutton J. E. Dukerich J. M. (1991). Keeping an eye on the mirror: image and identity in organizational adaptation. *Acad. Manag. J.* 34 517–554. 10.2307/256405

[B16] Eagly A. H. Karau S. J. (2002). Role congruity theory of prejudice toward female leaders. *Psychol. Rev.* 109 573–598. 10.1037/0033-295X.109.3.573 12088246

[B17] Freire P. (1973). *Education for Critical Consciousness.* New York, NY: Continuum.

[B18] Garcia-Retamero R. Lopez-Zafra E. (2006). Prejudice against women in male- congenial environments: perceptions of gender role congruity in leadership. *Sex Roles* 55 51–61. 10.1007/s11199-006-9068-1

[B19] Gladman K. Lamb M. (2012). *Women on Boards Survey.* Available at: http://www.boardagender.org/files/GMI-Ratings-2012-Women-on-Boards-Survey-F.pdf

[B20] Glaser B. G. Strauss A. L. (1967). *The Discovery of Grounded Theory: Strategies for Qualitative Research.* Chicago, IL: Aldine.

[B21] Hill C. Corbett C. St. Rose A. (2010). *Why So Few? Women in Science, Technology, Engineering, and Mathematics.* Washington, DC: American Association of University Women (AAUW).

[B22] Hughes R. M. (2010). *The Process of Choosing Science, Technology, Engineering, and Mathematics Careers by Undergraduate Women: A Narrative Life History Analysis.* Ph.D. dissertation. Florida State University Libraries, Tallahassee, FL.

[B23] Jacobs J. A. (1992). Women’s entry into management: trends in earnings, authority, and values among salaried managers. *Adm. Sci. Q.* 37 282–301. 10.2307/2393225

[B24] Kaschak E. (1978). Sex bias in student evaluations of college professors. *Psychol. Women Q.* 2 235–242. 10.1111/j.1471-6402.1978.tb00505.x

[B25] Kelly E. (1999). Theorizing corporate family policies: how advocates built the ‘business case’ for ‘family-friendly’ programs. *Res. Sociol. Work* 7 1169–1202.

[B26] Kreiner G. E. Hollensbe E. C. Sheep M. L. (2009). Balancing borders and bridges: negotiating the work-home interface via boundary work tactics. *Acad. Manag. J.* 52 704–730. 10.5465/AMJ.2009.43669916

[B27] Lincoln Y. S. Guba E. G. (1985). *Naturalistic Inquiry.* Newbury Park, CA: Sage.

[B28] Locke K. (2001). *Grounded Theory in Management Research.* London: Sage.

[B29] McCullough L. (2011). Women’s leadership in science technology, engineering and mathematics: Barriers to participation. *Forum Public Policy* 2011 1–11.

[B30] Moss-Racusin C. A. Dovidio J. F. Brescoll V. L. Graham M. J. Handelsman J. (2012). Science faculty’s subtle gender biases favor male students. *Proc. Natl. Acad. Sci. U.S.A.* 109 16474–16479. 10.1073/pnas.1211286109 22988126 PMC3478626

[B31] Murphy M. C. Steele C. M. Gross J. J. (2007). Signaling threat: how situational cues affect women in math, science, and engineering settings. *Psychol. Sci.* 18 879–885. 10.1111/j.1467-9280.2007.01995.x 17894605

[B32] National Science Foundation (2015a). *Doctorate Degrees Awarded to Women.* Available at: http://www.nsf.gov/statistics/2015/nsf15311/tables/pdf/tab7-2.pdf

[B33] National Science Foundation (2015b). *Women, Minorities, and Persons with Disabilities in Science and Engineering.* Available at: http://www.nsf.gov/statistics/2015/nsf15311/digest/theme5.cfm#trends

[B34] Packard B. W. Gagnon J. L. LaBelle O. Jeffers K. Lynn E. (2011). Women’s experiences in the STEM community college transfer pathway. *J. Women Minor. Sci. Eng.* 17 129–147. 10.1615/JWomenMinorScienEng.2011002470

[B35] Portes A. (1998). Social capital: its origins and applications in modern sociology. *Ann. Rev. Sociol.* 24 1–24. 10.1146/annurev.soc.24.1.1

[B36] Richter J. (1984). *The Daily Transition between Professional and Private Life.* doctoral dissertation. Boston University Boston, MA.

[B37] Riffle R. Schneider T. Hillard A. Polander E. Jackson S. DesAutels P. (2013). A mixed methods study of gender, STEM department climate, and workplace outcomes. *J. Women Minor. Sci. Eng.* 19 227–243. 10.1615/JWomenMinorScienEng.2013005743

[B38] Sachdev I. Bourhis R. Y. (1991). Power and status differentials in minority and majority group relations. *Eur. J. Soc. Psychol.* 21 1–24. 10.1002/ejsp.2420210102

[B39] Schein E. H. (1971). The individual, the organization, and the career: a conceptual scheme. *J. Appl. Behav. Sci.* 7 401–426. 10.1177/002188637100700401

[B40] Shettle C. S. Roey J. Mordica R. Perkins C. Nord J. Teodorovic J. (2007). *The Nation’s Report Card: America’s High School Graduates: Results from the 2005 NAEP High School Transcript Study.* (NCES 2007-467) Washington, DC: U.S. Department of Education, National Center for Education Statistics.

[B41] Smith J. L. White P. H. (2002). An examination of implicitly activated, explicitly activated, and nullified stereotypes on mathematical performance: it’s not just a woman’s issue. *Sex Roles* 47 179–191. 10.1023/A:1021051223441

[B42] Sprague J. Massoni K. (2005). Student evaluations and gendered expectations: what we can’t count can hurt us. *Sex Roles* 53 779–793. 10.1007/s11199-005-8292-4

[B43] Strauss A. Corbin J. (1998). *Basics of Qualitative Research.* Thousand Oaks, CA: Sage.

[B44] Tower L. E. Dilks L. M. (2015). Work/life satisfaction policy in ADVANCE universities: assessing levels of flexibility. *J. Divers. High. Educ.* 8 157–174. 10.1037/a0039372

[B45] Trower C. A. Chait R. P. (2002). Faculty diversity: too little for too long. *Harv. Mag.* 104 33–38.

[B46] United States Census Bureau (2010). *Census Bureau Reports Nearly 6 in 10 Advanced Degree Holder Age 25-29 are Women.* Available at http://www.census.gov/newsroom/releases/archives/education/cb10-55.html

[B47] Valian V. (1999). *Why So Slow? The Advancement of Women.* Cambridge, MA: MIT Press.

[B48] Van Maanen J. (1982). “Boundary crossings: major strategies of organizational socialization and their consequences,” in *Career Issues in Human Resource Management* ed. Katz R. (Englewood Cliffs, NJ: Prentice-Hall) 85–115.

[B49] Wang C. Burris M. A. (1997). Photovoice: concept, methodology, and use for participatory needs assessment. *Health Educ. Behav.* 24 369–387. 10.1177/109019819702400309 9158980

[B50] Wang C. C. (1999). Photovoice: a participatory action research strategy applied to women’s health. *J. Womens Health* 8 185–192. 10.1089/jwh.1999.8.185 10100132

[B51] Weiler K. (1988). *Women Teaching for Change: Gender, Class, and Power.* Westport, CT: Bergin & Garvey.

[B52] Welch J. L. Wiehe S. E. Palmer-Smith V. Dankoski M. E. (2011). Flexibility in faculty work-life policies at medical schools in the Big Ten conference. *J. Womens Health* 20 725–732. 10.1089/jwh.2010.2553 21501084

